# Ammonia Volatilization from Urea-Application Influenced Germination and Early Seedling Growth of Dry Direct-Seeded Rice

**DOI:** 10.1100/2012/857472

**Published:** 2012-02-01

**Authors:** Xiaoli Qi, Wei Wu, Farooq Shah, Shaobing Peng, Jianliang Huang, Kehui Cui, Hongyan Liu, Lixiao Nie

**Affiliations:** Crop Physiology and Production Center (CPPC), National Key Laboratory of Crop Genetic Improvement, MOA Key Laboratory of Crop Physiology, Ecology and Cultivation (The Middle Reaches of Yangtze River), Huazhong Agricultural University, Hubei, Wuhan 430070, China

## Abstract

Poor seed germination and early seedling growth associated with urea-induced soil ammonia volatilization are major constraints in the adoption of dry direct-seeded rice. To directly examine soil ammonia volatilization and its damage to seed germination and early seedling growth of dry direct-seeded rice when urea is applied at seeding, two Petri-dish incubation experiments and a field experiment were conducted. Ammonia volatilization due to urea application significantly reduced seed germination and early seedling growth of dry direct-seedling rice. NBPT significantly reduced ammonia volatilization following urea application. The application of ammonium sulfate, instead of urea at seeding, may mitigate poor crop establishment of dry direct-seeded rice. Root growth of dry direct-seeded rice was more seriously inhibited by soil ammonia volatilization than that of shoot. Results suggest that roots are more sensitive to soil ammonia toxicity than shoots in dry direct-seeded rice system when N is applied as urea at seeding.

## 1. Introduction

Increasing scarcity of fresh-water resources for agriculture in many areas threatens the sustainability of the irrigated rice production systems. By 2025, 17 million ha of irrigated rice areas may experience “physical water scarcity,” and 22 million ha in South and Southeast Asia may suffer “economic water scarcity” [1]. Water-saving technologies with reduced water usage and increased water-use efficiency can greatly contribute in this regard.

A technology, “dry direct-seeded rice” has been developed to reduce water input, save labor demand, and increase water productivity. The definition of dry direct-seeded rice is that dry seed is broadcast onto soil surface and then incorporated either by ploughing or harrowing while the soil is still dry [2]. Direct-seeded rice avoids soil puddling and maintains continuous moist soil conditions and thus reduces the overall water demand for rice culture. Nearly 15% of all lowland rice is direct seeded [2]. In South Asia, direct-seeded rice is being practiced on terraced and sloppy lands of Bangladesh, along the coast and Western Himalayan region of India [3]. Direct-seeding technology is also adopted in upland rice [4], aerobic rice [5], and rice on raised beds [6]. Dry seeding with subsequent aerobic soil conditions on raised beds avoids water required during land preparation and thus reduces overall water demand [7].

Dong et al. [8] reported that dry direct-seeded rice achieved grain yield of 11.2 t ha^−1^ in Zhejiang province. In northeastern China, dry direct-seeded rice had on average 5.33% higher grain yields and about 25–50% lower water use than conventionally transplanted rice [9]. Zhu [10] documented that dry direct-seeded rice increased the grain yield by 22% and reduced the water input by 6,000 m^3^ ha^−1^ compared with traditional rice cultivation. In India, dry direct-seeded rice on raised beds was reported to save about 37–40% of irrigation water with only 5-6% of variation in grain yield [11]. Input water savings of 35–57% have been reported for dry seeded rice sown into nonpuddled soil compared with continuously flooded soil [12, 13]. Results of farmers' participatory trials suggested a small increase or 10% decline in yield of dry direct-seeded rice on the flat bed compared with puddled transplanted rice, and around 20% reduction in irrigation time or water use [14, 15].

However, poor seed germination and early-seedling growth are major constraints in the adoption of dry direct-seeded rice. The possible causes include improper soil water content, unsuitable seeding depth, and ammonia toxicity due to urea application. Ammonia toxicity induced by urea application has been reported in wide range of crops, such as, maize, wheat, barley, oats, sorghum, and rye [16–19]. In dry direct-seeded rice system, the toxicity of ammonia volatilization may be one of the main causes responsible for poor seed germination and early seedling growth, when urea fertilizer is applied at seeding [20–22].

Urea is the most widely used N fertilizer in the world because of its high solubility, high N content, low cost, and ease in handling and accounts for over 50% of all N applied [23]. Upon application to soil, urea is rapidly hydrolyzed by urease enzymes to form ammonia, and the accumulation of excess ammonia can result in toxicity to plants [24, 25]. The application of urease inhibitor is generally believed to be a promising way to reduce soil ammonia volatilization associated with urea application and to improve the efficiency of urea. Previous research reported that NBPT is superior to PPD (phenyl phosphorodiamidate) and HQ (hydroquinone) in retarding urea hydrolysis and reducing NH_3_ volatilization [26, 27]. Our objectives were (1) to examine the soil ammonia volatilization and its subsequent effects on seed germination and early seedling growth of dry direct-seeded rice under urea, urea + NBPT, and ammonium sulfate applied at seeding, (2) to identify the effectiveness of NBPT and ammonium sulfate in reducing soil ammonia volatilization and improving seed germination and early seedling growth of dry direct-seeded rice.

## 2. Materials and Methods

### 2.1. Petri dish Incubation Experiment

Soil ammonia volatilization from urea application and its effects on seed germination and early seedling growth of dry direct-seeded rice were examined in two Petri dish incubation experiments. Soils for Petri dish incubation experiments were collected from the top 25 cm of three fields, where tea plant (Soil I), wheat (Soil II), and vegetables (Soil III) were grown, respectively, in Hubei province. Physical and chemical properties of the three soil samples are listed in Table 1. Before filling the Petri dish, the soil was air-dried, pulverized, crushed to pass through a 2 mm sieve, and well mixed. Two sizes of plastic Petri dishes with a diameter of 5.5 and 14.5 cm were used. A 150 g of air-dried soil from each soil sample was placed in a larger plastic Petri dish. 

In both Petri dish incubation experiments, the N rate was 0.15 g N per Petri dish and NBPT was applied at 1.00% on a urea basis. Urea, ammonium sulfate, and NBPT were applied to the soil in each Petri dish in solution, and the total volume of solution was kept as 50 mL. To simulate soil water content suitable for direct-seeded rice, soil inside the Petri dishes was kept under aerobic conditions.

In Petri dish incubation experiment 1, ammonia volatilization was measured using the method of Conway microdiffusion incubation adapted for soil by Bremner and Krogmeier [17]. A smaller uncovered Petri dish with diameter of 5.5 cm containing 10 mL of 2% boric acid and 1-2 drops of mixture indicators (bromocresol green and methyl red) was placed in the center of the larger Petri dish to serve as a trap for ammonia gas. Then the lid of the larger Petri dish was covered and sealed so that the ammonia gas could be trapped by the boric acid without gas leakage from this system.

Petri dishes were placed inside a darkened growth chamber for 4 d at 30°C and 70% humidity using a completely randomized design. Each treatment was replicated four times with four Petri dishes. The amount of ammonia volatilized from soil was determined by titrating the boric acid using 0.01 N HCl [28].

In experiment 2, the treatments and incubation methods were the same to those of Petri dish incubation experiment 1. A smaller uncovered Petri dish containing 10 presoaked seeds of Apo (an upland rice variety) with moistened filter paper was placed in the center of a larger Petri dish, instead of boric acid. Inside the sealed Petri dish, the presoaked seeds were exposed to ammonia volatilized, but without any contact with the soil. The Petri dish was incubated under the same conditions used in Petri dish incubation experiment 1. The fresh shoot and root were weighed and recorded, and root tip number was counted using a WinRHIZO scanner (Regeant Instrument, Quebec, Canada).

### 2.2. Field Experiment

A field experiment was conducted at the experimental station of Zhougan village, Dajin County, Hubei Province, China (29°51′ N 115°33′ E). The soil from this field experiment was named as soil IV. An improved upland rice variety, Apo and a newly bred hybrid rice variety with tolerance to drought, Hanyou3 were used. Treatments were 0 N, U50 (urea, 50 kg N ha^−1^), U100 (urea, 100 kg N ha^−1^), U50 + NBPT1 (50 kg N ha^−1^ + 1.09 kg NBPT ha^−1^), U100 + NBPT2 (100 kg N ha^−1^ + 2.18 kg NBPT ha^−1^), AS50 (50 kg N ha^−1^), and AS100 (100 kg N ha^−1^) and arranged in a randomized complete block design with four replications. Each plot area was 2.6 m^2^ (2 m × 1.3 m) and divided into two subplots to accommodate both Apo and Hanyou3. Plots were dry ploughed and harrowed during land preparation. Dry seeds were directly sown in furrow manually with row spacing of 25 cm on 23 July 2010. The seeding rate for both Apo and Hanyou3 was 80 kg ha^−1^. Immediately after sowing, urea or ammonium sulfate was placed near the seed rows. Urease inhibitor, NBPT, was sprayed in soil uniformly in solution. The sown seeds and applied fertilizers were then covered with soil. Soil was kept wet for one week after sowing to promote good germination and early seedling growth after which the field was kept under rainfed conditions. Pests, diseases, and weeds were intensively controlled. 

In accordance with the Petri dish incubation experiment, soil ammonia volatilization in the field was also evaluated using the method of Conway microdiffusion incubation adapted for soil by Bremner and Krogmeier [17]. An airproof container with diameter of 15 cm was put on the soil surface, inside which a small uncovered Petri dish with boric acid was put. A wooden board was placed on the container to avoid gas leakage from the system, to provide shade so that the inner temperature is kept minimum, and to support the container so that the strong wind may not displace it. The ammonia volatilized from soil was trapped by 2% boric acid and then titrated by 0.01 N HCl every day since one day after sowing, until the amount of ammonia volatilized from soil was negligible [28]. Total ammonia volatilization was the sum of the ammonia volatilized each day. Seed germination percentage was examined by counting the plants emerged from one meter along the row at 10 days after sowing.

Data were analyzed following analysis of variance [29], and mean comparison between treatments was performed based on the Least Significant Difference (LSD) test at the 0.05 probability level.

## 3. Results

Soils II and IV were near neutral soil, while soils I and III were acidic and alkaline, respectively (Table 1). Soil IV contained more organic carbon and total N than soils I, II, and III, and had highest cation exchange capacity of all the soils in this study. However, soil IV contained less available K than any other soil used in our study. Soil II had the highest Olsen P and available K among all soils. Soils I and IV contained the least sand and clay, respectively, compared with other soils.

For all three soils in experiment 1, ammonia volatilization was negligible in the Petri dishes with ammonium sulfate at 0.15 g N per Petri dish and the zero N input control Petri dishes (Table 2). Ammonia volatilization was markedly increased by application of urea at 0.15 g N per Petri dish, and NBPT reduced it significantly. In both soils I and II, ammonia volatilization by application of urea at 0.15 g N per Petri dish was above 4000 *μ*g per perti dish, while ammonia volatilization was only 1785 *μ*g per perti dish in soil III. The inhibitory effect of NBPT on ammonia volatilization was greater in soil II than the other two soils.

In experiment 2, application of urea to soils I–III at 0.15 g N per Petri dish significantly decreased parameters of root growth compared with zero N input control (Table 3). In soils I and II, the shoot growth was not inhibited by urea application, while shoot growth was increased by urea application in soil III. NBPT significantly alleviated the inhibitory effect on root growth due to urea application in all three soils but did not improve the shoot growth. Root growth was better under ammonium sulfate application than urea in soils I and II; however, there was no difference between ammonium sulfate and urea application in soil III. Urea + NBPT improved the plant growth up to the level of ammonium sulfate application or even exceeded the level of ammonium sulfate. The seed germination percentage was reduced by application of urea, and NBPT reversed the reduction; however, the differences were not statistically significant.

**Figure 1:**
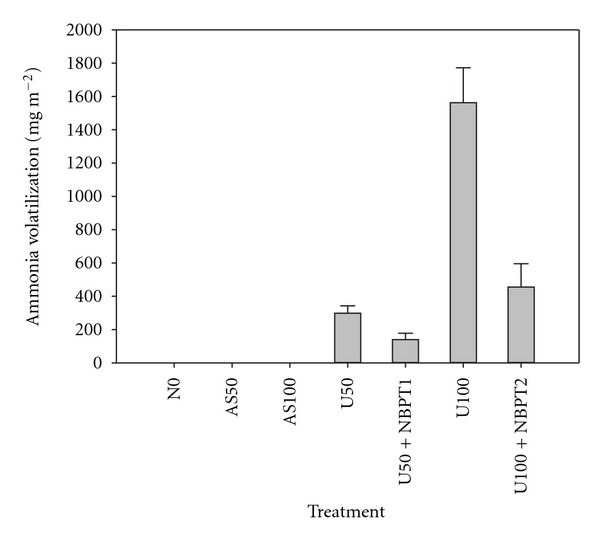
Ammonia volatilization under application of AS50 (ammonium sulfate, 50 kg N ha^−1^), AS100 (ammonium sulfate, 100 kg N ha^−1^), Urea50 (50 kg N ha^−1^), Urea100 (100 kg N ha^−1^), Urea50 + NBPT1 (50 kg N ha^−1^ + 1.09 kg NBPT ha^−1^), and Urea100 + NBPT2 (100 kg N ha^−1^ + 2.18 kg NBPT ha^−1^) in comparison with zero N input (control) in a field experiment. Ammonia volatilization was the sum of ammonia volatilized during 14 days after sowing. Error bars represent the standard error.

**Figure 2:**
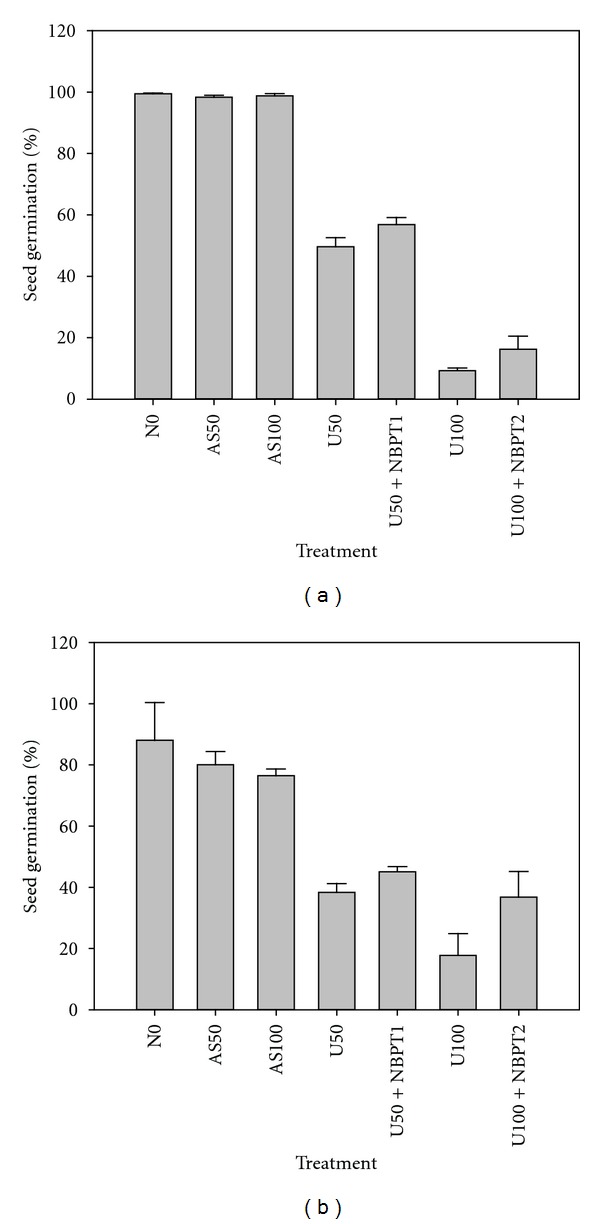
Seed germination percentage of Apo (a) and Hanyou3 (b) under application of AS50 (ammonium sulfate, 50 kg N ha^−1^), AS100 (ammonium sulfate, 100 kg N ha^−1^), U50 (urea, 50 kg N ha^−1^), U100 (urea, 100 kg N ha^−1^), U50 + NBPT1 (50 kg N ha^−1^ + 1.09 kg NBPT ha^−1^), and U100 + NBPT2 (100 kg N ha^−1^ + 2.18 kg NBPT ha^−1^) in comparison with zero N input (control) in a field experiment. Error bars represent the standard error.

In the field experiment, ammonia volatilization was negligible in treatments of 0N, AS50, and AS100, but ammonia volatilization was significantly increased by application of urea and was reduced by NBPT (Figure 1). Ammonia volatilization increased as urea rates increased from 50 to 100 kg N ha^−1^. Especially in the treatment of U100, ammonia volatilization was 1562 mg m^−2^, while application of NBPT reduced ammonia volatilization to 455 mg m^−2^. Seed germination percentages of both Apo and Hanyou3 were significantly decreased by urea application compared with zero N input control and decreased as the rate of urea increased from 50 to 100 kg N ha^−1^ (Figures 2(a) and 2(b)). Application of NBPT significantly mitigated the reduction in seed germination percentage for both rice varieties. In the treatment of U100, seed germination percentage of Apo was 9.32%, and application of NBPT increased seed germination percentage to 16.22% (Figure 2(a)); while for Hanyou3 seed germination percentage was 17.76% and increased to 36.81% by application of NBPT (Figure 2(b)). Application of ammonium sulfate did not significantly affect seed germination. When N was applied as urea at 100 kg N ha^−1^, the seed germination percentage of Hanyou3 was higher than that of Apo, suggesting that Hanyou3 was more tolerant to ammonia toxicity than Apo in dry direct-seeding systems. 

## 4. Discussion

Dry direct-seeded rice suffers from ammonia toxicity when urea is applied at seeding. Application of NBPT significantly decreased soil ammonia volatilization and improved the root growth of rice seedlings in dry direct-seeding system as compared to without NBPT (Tables 2 and 3). However, shoot growth did not respond to NBPT or was reduced. The symptoms of ammonia toxicity following urea application lead to reduced germination, root damage, and poor seedling growth [16, 17, 20], which are confirmed in this study. In an open field experiment, seed germination percentage of rice variety Apo and Hanyou3 was reduced to 9.32% and 17.76%, respectively, when urea fertilizer was applied at 100 kg N ha^−1^ (Figure 2). In Petri dish incubation experiment 2, the growth of shoot and root was adversely affected by ammonia volatilization (Table 3). However, two evidences from this study suggest that roots of dry direct-seeded rice are more sensitive to ammonia toxicity than the shoots. First, root growth of dry direct-seeded rice is more seriously inhibited by ammonia volatilization than that of shoot (Table 3). Second, application of NBPT significantly improved parameters of root growth, but not those of shoot (Table 3).

In Petri dish and open field experiments, urea reduced seed germination and root growth of seedling while ammonium sulfate caused no adverse effects. Upon the application of urea to soil, urea is rapidly hydrolyzed to ammonia by urease enzymes. The reaction of hydrolysis results in a short-term increase in soil pH close to the location where urea fertilizer is applied [24, 25]. Then accumulation of excess ammonia under alkaline soil conditions induces ammonia toxicity. While acidifying N fertilizers, such as ammonium sulfate and diammonium phosphate, do not undergo alkaline hydrolysis and thus are less likely to form excessive amount of ammonia [22, 30].

In the field experiment, seed germination percentage was significantly reduced by application of urea (Figures 2(a) and 2(b)). However, the seed germination percentage was statistically the same among the treatments of urea, ammonium sulfate, and urea + NBPT, and the zero N input control in Petri dish incubation experiment 2 (Table 3). The reason could be that in Petri dish incubation experiments the seeds were presoaked for 24 hrs before they were exposed to ammonia volatilized, while dry seeds were directly sown to soil in the field experiment, suggesting that ammonia toxicity to seed germination might start during water swelling of dry seeds.

To minimize the adverse effects of ammonia volatilization, appropriate N management strategies should be considered. In addition to application of urease inhibitors, some other practical options, such as delaying the first urea application until two weeks after emergence, optimizing the timing and placement of urea, using slow-released N fertilizers [31], splitting the application of urea into three or more doses, banding urea so that the granules are not placed too close to seeds rows, and deep placement of urea [21, 32] should be adopted.

In this study, the N rate in these two Petri dish incubation experiments is aimed to examine the soil ammonia volatilization and its toxicity to seed germination and early seedling growth of dry direct-seeded rice if seeds are sown close to concentrated fertilizer bands and does not reflect typical field application rate when calculated at the aggregate scale. The effects of soil ammonia volatilization on seed germination and early seedling growth of dry direct-seeded rice under urea application at seeding were described. However, the possible mechanisms of ammonia toxicity have not been addressed. The physiological mechanisms responsible for the toxicity of ammonia to seed germination and root growth of dry direct-seeded rice will be studied in the future.

## 5. Conclusion

Ammonia volatilization from urea at seeding resulted in poor seed germination and early seedling growth of dry direct-seeded rice. Urease inhibitor, NBPT, significantly alleviated ammonia volatilization following urea application. The application of ammonium sulfate, instead of urea at seeding, may mitigate poor crop establishment of dry direct-seeded rice. Findings from this study suggest that roots of dry direct-seeded rice are more sensitive to ammonia toxicity than the shoots when urea is applied at seeding. Physiological mechanisms responsible for the toxicity of ammonia to seed germination and root growth of dry direct-seeded rice will be addressed in the future.

## Figures and Tables

**Table 1:** Chemical and physical properties of the soils used in Petri dish incubation and field experiments.

Parameter	Petri dish experiment			Field experiment	Ave. S.E
	Soil I	Soil II	Soil III	Soil IV	
pH	4.8	7.3	7.9	6.5	0.02
Organic C (g/kg)	10.5	10.2	7.0	16.2	0.13
Total N (g/kg)	1.35	1.24	1.02	1.98	0.015
Olsen P (mg/kg)	8.4	24.0	9.7	18.3	0.31
Available K (mg/kg)	89	150	108	62	0.2
CEC (cmol_c_/kg)	8.5	11.0	9.3	49.9	0.18
Clay (%)	23	30	17	10	0.95
Silt (%)	74	46	70	64	0.88
Sand (%)	3	24	13	26	0.55

Soil I, II, and III were collected from fields where tea plant, wheat, and vegetables were grown, respectively.

Soil IV was the field experiment soil in this study.

**Table 2:** Ammonia volatilization from three different soils with ammonium sulfate, urea, and urea + NBPT in comparison with zero N input (control) in Petri dish incubation experiment.

Soil source	Ammonia volatilization^a^ (*μ*g per Petri dish)			
	Control	AS^b^	Urea^b^	Urea^b^ + NBPT^c^
Soil I	0 c	0 c	4029 a	323 b
Soil II	0 b	0 b	4250 a	0 b
Soil III	0 c	0 c	1785 a	136 b

Within a row, means followed by different letters are significantly different at 0.05 probability level according to Least Significant Difference (LSD) test.

^a^Ammonia volatilization was determined at four days after incubation.

^b^The rate of ammonium sulfate and urea was 0.15 g N per Petri dish.

^c^The rate of NBPT was 1.5 mg per Petri dish.

**Table 3:** Seed germination percentage, shoot fresh weight, root fresh weight, and root tip number of rice variety Apo grown in three different soils with ammonium sulfate, urea, and urea + NBPT in comparison with zero N input (control) in Petri dish incubation experiment 2. The presoaked seeds were not in contact with soil but exposed to ammonia gas volatilized from soils.

Parameter	Control	AS^a^	Urea^a^	Urea^a^ + NBPT^b^
*Soil I *				
Seed germination percentage (%)	100 a	98 a	90 a	100 a
Shoot FW (g per Petri dish)	0.16 a	0.17 a	0.15 a	0.15 a
Root FW (g per Petri dish)	0.17 a	0.14 b	0.01 c	0.15 ab
Root tip number per Petri dish	22 b	32 a	2 c	23 b

*Soil II*				
Seed germination percentage (%)	100 a	98 a	94 a	100 a
Shoot FW (g per Petri dish)	0.15 a	0.17 a	0.15 a	0.17 a
Root FW (g per Petri dish)	0.14 a	0.12 a	0.05 b	0.15 a
Root tip number per Petri dish	27 a	26 a	5 b	23 a

*Soil III*				
Seed germination percentage (%)	100 a	94 a	98 a	98 a
Shoot FW (g per Petri dish)	0.14 b	0.16 b	0.19 a	0.16 b
Root FW (g per Petri dish)	0.12 b	0.07 c	0.07 c	0.15 a
Root tip number per Petri dish	24 b	7 c	5 c	27 a

Within a row, means followed by different letters are significantly different at 0.05 probability level according to Least Significant Difference (LSD) test.

^a^The rate of ammonium sulfate and urea was 0.15 g N per Petri dish.

^b^The rate of NBPT was 1.5 mg per Petri dish.
